# Serum amyloid A (SAA): a novel biomarker for uterine serous papillary cancer

**DOI:** 10.1038/sj.bjc.6605129

**Published:** 2009-06-16

**Authors:** E Cocco, S Bellone, K El-Sahwi, M Cargnelutti, F Casagrande, N Buza, F A Tavassoli, E R Siegel, I Visintin, E Ratner, D-A Silasi, M Azodi, P E Schwartz, T J Rutherford, S Pecorelli, A D Santin

**Affiliations:** 1 Department of Obstetrics, Gynecology and Reproductive Sciences, Yale University School of Medicine, New Haven, CT, USA; 2 Department of Pathology, Yale University School of Medicine, New Haven, CT, USA; 3 Department of Biostatistics, University of Arkansas for Medical Sciences, Little Rock, AR, USA; 4 Division of Gynecologic Oncology, University of Brescia, Brescia, Italy

**Keywords:** uterine serous papillary cancer, serum amyloid A, biomarkers, endometrial carcinoma, tumour markers

## Abstract

**Background:**

Uterine serous papillary carcinoma (USPC) is a biologically aggressive variant of endometrial cancer. We investigated the expression of Serum Amyloid A (SAA) and evaluated its potential as a serum biomarker in USPC patients.

**Methods:**

SAA gene and protein expression levels were evaluated in USPC and normal endometrial tissues (NEC) by real-time PCR, immunohistochemistry (IHC), flow cytometry and by a sensitive bead-based immunoassay. SAA concentration in 123 serum samples from 51 healthy women, 42 women with benign diseases, and 30 USPC patients were also studied.

**Results:**

SAA gene expression levels were significantly higher in USPC when compared with NEC (mean copy number by RT–PCR=162 *vs* 2.21; *P*=0.0002). IHC revealed diffuse cytoplasmic SAA protein staining in USPC tissues. High intracellular levels of SAA were identified in primary USPC cell lines evaluated by flow cytometry and SAA was found to be actively secreted *in vitro*. SAA concentrations (*μ*g ml^−1^) had a median (95% CIs) of 6.0 (4.0–8.9) in normal healthy females and 6.0 (4.2–8.1) in patients with benign disease (*P*=0.92). In contrast, SAA values in the serum of USPC patients had a median (95% CI) of 15.6 (9.2–56.2), significantly higher than those in the healthy group (*P*=0.0005) and benign group (*P*=0.0006). Receiver operating characteristics (ROC) analysis of serum SAA to classify advanced- and early-stage USPC yielded an area under the ROC curve of 0.837 (*P*=0.0024).

**Conclusion:**

SAA is not only a liver-secreted protein but is also a USPC cell product. SAA may represent a novel biomarker for USPC to assist in staging patients preoperatively, and to monitor early-disease recurrence and response to therapy.

Cancer of the uterine corpus is the most prevalent gynaecological tumour in women, with an estimated 40 100 cases and 7470 deaths in the United States in 2008 (Jemal *et al*, 2008). Two subtypes of endometrial carcinoma, namely type I and type II tumours, have been described based on both clinical and histopathological variables (Bohkman, 1983). Type I endometrial cancers, which account for the majority of the cases, are usually well differentiated and endometrioid in histology. These neoplasms are frequently diagnosed in younger women, are associated with a history of hyperestrogenism as the main risk factor, and typically have a favourable prognosis with appropriate therapy. In contrast, type II endometrial cancers are poorly differentiated tumours, often with serous papillary (USPC) or clear cell histology. Although USPC account for only a minority of endometrial cancers (i.e., less than 10%), about 40% of all relapses occur in this group of patients (Hendrickson *et al*, 1982; Goff *et al*, 1994; Carcangiu and Chambers, 1995; Chan *et al*, 2003). The discovery of novel diagnostic and therapeutic markers against this aggressive subset of endometrial cancers remains a high priority.

High-throughput genomic analysis represents a new tool for the discovery of novel molecular tumour markers. Using this technology, our group has recently evaluated the genetic fingerprints of USPC (Santin *et al*, 2004, 2005). Among the several candidate target genes identified, the gene encoding for human serum Amyloid A (SAA), an HDL-associated lipoprotein known to have a major role as a modulator of inflammation and in the metabolism and transport of cholesterol (Yamada, 1999; Malle *et al*, 2009), was consistently found as one of the top upregulated genes in USPC. In humans the SAA gene family consists of three genes (SAA1, SAA2, and SAA4) and a (pseudo) gene (SAA3) (Malle *et al*, 2009), all clustered on the short arm of chromosome 11. SAA1 and SAA2 genes, referred to as acute-phase genes share approximately 95% overall sequence identity in their promoter regions, exons, and introns (Uhlar *et al*, 1994). Earlier studies have shown an association between high SAA concentration and various human malignancies (Skinner *et al*, 1979; Yamada, 1999). However, it is only recently that SAA has been proposed as a potentially useful biomarker to monitor patients harbouring human tumours including gastric and nasopharyngeal cancer (Cho *et al*, 2004; Chan *et al*, 2007). Moreover, in lung cancer patients, using mass spectrometry and proteomic technologies, SAA was identified as the top differentially expressed protein able to differentiate the serum of patients from the serum of healthy individuals (Howard *et al*, 2003).

One major problem with the use of SAA, an acute-phase reactant, as a potential serum marker in human cancer patients, is the fact that its elevation in the serum of patients is suggested to be of liver origin rather than a tumour–cell product (Diamandis, 2004). Indeed, SAA level in the blood may elevate up to 1000-fold when the body responds to various injuries including trauma and various inflammations in addition to neoplasia (Diamandis, 2004). Importantly, however, extrahepatic SAA expression has been previously demonstrated in several histologically normal tissues, predominantly by their epithelium (Urieli-Shoval *et al*, 1998, 2000). Unfortunately, only scant information regarding SAA expression in malignant human tissues has been so far reported and, to our knowledge, no studies have yet addressed a potential direct secretion of SAA by human endometrial tumours. This report represents the first investigation examining SAA 1 expression and secretion in human serous papillary endometrial carcinoma, the most aggressive variant of endometrial cancer.

## Patients and methods

### Primary tumours

Fresh tumour samples were derived from primary specimens staged according to the FIGO operative staging system. Only specimens with >75% tumour content were used in the RT–PCR experiments. Briefly, fresh tumour biopsies from 16 USPC (obtained from seven Caucasian and nine African-American patients age 65±8: mean±s.d.) were obtained under approval of the Institutional Review Board at the time of surgery and analysed for SAA expression. Patient characteristics from which tumour biopsies were obtained included 2 stage I, 7 stage III and 7 stage IV patients. Total abdominal hysterectomy, bilateral salpingo-oophorectomy and lymph node dissection were performed in all endometrial cancer patients. Normal endometrial control cell samples (NEC) were obtained from biopsies of benign hysterectomy specimens obtained from women of similar age. Three primary USPC cell lines (i.e., USPC-ARK-1, USPC-ARK-2, USPC-ARK-3) were also established as short-term cultures following previously reported standard tissue culture techniques (Santin *et al*, 2004, 2005). RNA extraction was performed at a tumour–cell confluence of 50–80% after a minimum of two to a maximum of 20 passages *in vitro*. The epithelial nature and the purity of tumour cultures was verified by immunohistochemical staining and flow cytometric analysis with antibodies against cytokeratin and vimentin as previously described (Santin *et al*, 2004, 2005). Only primary cultures which had at least 90% viability and contained >99% epithelial cells were used for SAA quantification by a sensitive bead-based immunoassay, as described below.

### RNA extraction and quantitative real-time PCR

RNA isolation from all primary snap frozen samples including 16 primary USPC as well as three normal endometrial cell controls was performed using TRIzol Reagent (Invitrogen, Carlsbad, CA, USA) according to the manufacturer's instructions. Quantitative PCR was done with a 7500 Real-Time PCR System using the manufacturer's recommended protocol (Applied Biosystems, Foster City, CA, USA) to evaluate the expression of SAA in all the samples. Each reaction was run in triplicate. Briefly, 5 *μ*g of total RNA from each sample was reverse transcribed using SuperScript III first-strand cDNA synthesis (Invitrogen). Five microliters of reverse-transcribed RNA samples (from 500 *μ*l of total volume) were amplified by using the TaqMan Universal PCR Master Mix (Applied Biosystems) to produce PCR products specific for SAA. The primers for SAA were obtained from Applied Biosystems (Assay ID Hs00761940_s1). Owing to the approximately 95% sequence homology between SAA1 and SAA2, these primers are reported to recognise both forms of SAA. The comparative threshold cycle (*C*_t_) method (Applied Biosystems) was used to determine the gene expression in each sample relative to the value observed in the lowest nonmalignant endometrial epithelial cell sample, using glyceraldehyde-3-phosphate dehydrogenase (Assay ID Hs99999905_m1) RNA as internal controls.

### Intracellular flow cytometry

The mouse anti-human anti-SAA1 monoclonal antibody (i.e., clone mcl, DAKO Corporation; Carpinteria, CA), was used for our flow cytometry study. Briefly, freshly established USPC cell lines and control cells were fixed with 2% paraformaldehyde in PBS, washed and permeabilised by incubation in PBS plus 1% BSA and 0.5% saponin (S-7900, Sigma, St Louis, MO, USA) for 10 min at room temperature. Tumour cells were stained with anti-SAA MAb and isotype-matched controls (DAKO, Carpinteria, CA, USA). After staining, cells were washed twice with PBS plus 0.5% BSA. Secondary goat-anti-mouse antibody (IgG1-FITC, cat no. 349031, Beckton Dickinson, San Jose, CA, USA) was then added for 30 min at 4°C. Cells were then washed twice with PBS plus 0.5% BSA. Analysis was conducted with a FACScalibur utilising CellQuest software (Beckton Dickinson).

### SAA immunostaining of formalin-fixed tumour tissues

Formalin-fixed paraffin-embedded normal endometrial control tissues (five samples) and endometrial tumour tissues (eight samples) including the tumours from which the three primary USPC cell lines were established were evaluated by standard immunohistochemical staining for SAA expression. Study blocks were selected after histopathologic review by a surgical pathologist. The most representative block was selected for each specimen. Briefly, deparaffinised and rehydrated sections were treated according to the manufacturer's instructions (DAKO). The antibody was diluted 1 : 20 in DAKO Antibody Diluent and incubated at pH 9.0 for half an hour at room temperature. DAKO Envision system was used for secondary detection and colour was developed using DAB chromogen (DAKO) for 5 min followed by counterstaining with haematoxylin. The anti-SAA monoclonal antibody used (i.e., clone mcl, DAKO), was directed against SAA 1. The preparation and specificity of this antibody has been previously described and demonstrated (Gutfeld *et al*, 2006). Colour was developed using AEC substrate (Zymed Laboratories Inc., South San Francisco, CA, USA) for 10 min followed by counterstaining with haematoxylin. Negative controls included replacement of the primary antibodies by PBS and by normal mouse isotype-matched IgG (IgG2a, kappa; DAKO). Liver sections were used as positive controls. SAA intensity of staining was graded semi-quantitatively as 0 (negative=no staining), 1+ (focal, weak cytoplasmic staining), 2+ (diffuse weak, or focal moderate cytoplasmic staining), or 3+ (diffuse, strong cytoplasmic staining).

### Analysis of SAA secretion in tumour samples

To evaluate the potential secretion of SAA by primary USPC, supernatants obtained from USPC-ARK-1, USPC-ARK-2, and USPC-ARK-3 as well as multiple control cell lines including normal human fibroblasts, EBV-transformed B cells (LCL) and cervical carcinoma cell lines were evaluated by a sensitive bead-based immunoassay (Millipore Corp. Danvers, MA, USA). Briefly, tumour supernatants to be tested for SAA secretion were collected by primary tumour cell lines seeded at a density of 1 × 10^5^ cells per ml in tissue culture Petri dishes (Corning, Corning, NY, USA) in RPMI-1640 media, supplemented with 10% FBS (i.e., USPC and human fibroblasts), or serum-free keratinocyte medium (KFSM, i.e., cervical cancer cell lines). After 72 h incubation at 37°C, supernatants were aspirated, rendered cell-free by centrifugation at 1500 r.p.m. for 10 min, and stored at −20°C before being analysed for SAA by a bead-based immunoassay (see below).

### Measurement of SAA concentration in serum samples

SAA concentration was quantified in the serum of 51 apparently healthy women (age 59.6±10.9: mean±s.d.), 42 women with benign diseases (i.e., 22 uterine fibroids, 8 ovarian cysts and 12 endometrial polyps, age 51.2±15.6), and 30 women with histologically proven primary USPC (age 64.7±9.5), including seven stage I, four stage II, eight stage III and 11 stage IV patients, by a commercially available bead-based immunoassay (Lincoplex kit, acute-phase proteins, Millipore). In brief, the assay is based on conventional sandwich assay technology. The antibody specific to SAA is covalently coupled to Luminex microspheres. After a final wash, the beads are resuspended in buffer and read on a Bio-Rad Luminex^100^ Instrument to determine the concentration of SAA. All specimens were tested in replicate wells. Results are reported as the mean of the replicates. Serum samples from all patients were collected before surgery and stored at −80°C until analysis.

### Statistical analysis

For q-RT–PCR data, the right-skewing was removed by taking copy number ratios relative to the lowest-expressing NEC sample (‘relative copy numbers’), log_2_ transforming them to Δ*C*_t_s, and comparing the results through unequal variance *t*-test for the USPC *vs* NEC difference. Group means with 95% confidence limits (CIs) were calculated by computing them on the Δ*C*_t_s and then reverse-transforming the results to obtain means (95% CIs) of relative copy numbers. The analyses of differences among supernatants obtained from tumour cultures with different histologies, and among expression levels measured by flow cytometry and IHC, were performed using the Wilcoxon–Mann–Whitney (WMW) test. SAA serum concentrations among the different groups of patients (i.e., healthy controls, benign gynaecologic diseases, and USPC) were summarised as medians (with rank-based 95% CIs) and ranges, and compared for pairwise differences through receiver operating characteristic (ROC) analysis in conjunction with the WMW test. Within the USPC group, the ability of serum SAA to distinguish between advanced-stage (III–IV) and early-stage (I–II) disease was assessed in detail using ROC analysis and tested by WMW test. NCSS (Number Cruncher Statistical Systems, Kaysville, UT) was used for the ROC analyses, Excel (Microsoft Corp., Redmond, WA, USA) was used for generating graphs, and SPSS (SPSS Inc., Chicago, IL, USA) was used for all other statistical analyses. A 5% significance level was used for all statistical comparisons.

## Results

### SAA expression in snap frozen USPC by quantitative real-time PCR

**Figure 1:**
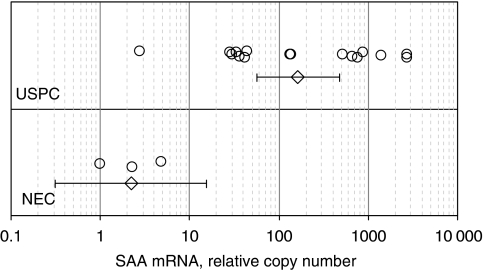
SAA mRNA copy number by quantitative RT–PCR in three normal endometrial control cell samples (NEC) and 16 uterine serous papillary carcinomas (USPC) snap frozen biopsies. The horizontal axis represents the relative number of copies compared with the lowest-expressing NEC (value of one). Circles denote individual observations, whereas diamonds with error bars represent group means with their 95% confidence intervals (CIs). Computation of means and 95% CIs are described in the text under Methods.

USPC are rare tumours, which may present in either pure forms, or admixed with endometrioid or clear cell tumour cells (i.e., mixed USPC). To minimise the risk of contamination of USPC RNA with that of normal cells or tumour cells with different histology, we extracted RNA to be evaluated for SAA expression by RT–PCR from 16 primary USPC with single-type differentiation (i.e., pure USPC). A comparison of the q-RT–PCR data for SAA in USPC *vs* NEC as controls is shown in Figure 1. Significant expression differences between USPC and NEC were readily apparent (Figure 1). Fifteen (94%) of the 16 USPC samples, compared with none (0%) of the three NEC samples, were found positive (>10) for SAA expression by RT–PCR. Relative copy numbers in NEC control samples had a mean (95% CI) of only 2.21 (0.315–15.5) and ranged from 1.00 to 4.80. In contrast, relative copies in USPC samples had a mean (95% CI) of 162 (55.8–470) and ranged from 2.76 to 2745. The fold change in mean relative copy numbers was 73.2 (Figure 1; *P*=0.0002).

### Intracellular SAA expression in USPC cell lines by flow cytometry

To determine whether the high expression of SAA gene detected by q-RT–PCR assays in flash frozen USPC also results in high expression of the SAA protein, we performed intracellular flow-cytometry analysis of SAA protein expression in three primary USPC established as short-term cultures *in vitro* in our laboratory. As shown in Table 1, all three primary USPC culture cell lines were found positive for intracellular SAA expression by flow cytometry (i.e., 100% positive cells; mean fluorescence intensity range from 40 to 77) (Table 1). In contrast, significantly lower expression of SAA was detected in Epstein–Barr transformed B cells (LCL) and cervical cancer cell lines (CVX) used as controls (i.e., 65–71% positive cells; mean fluorescence intensity range from 11 to 20 and 69–84% positive cells; mean fluorescence intensity range from 9 to 15, respectively) by flow cytometry (*P*=0.03 for both USPC *vs* LCL and USPC *vs* CVX, Table 1).

### SAA expression by immunohistochemistry in USPC

**Figure 2:**
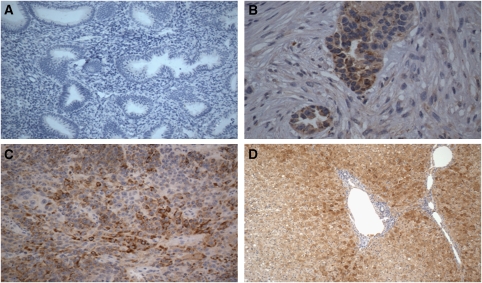
Representative immunohistochemical staining for SAA on NEC paraffin-embedded specimen (**A**, × 200), USPC-ARK-1 and USPC-ARK-2 (**B** and **C**, × 400), and a liver biopsy (**D**, × 100). NEC 1 showed negative staining for SAA whereas prominent cytoplasmic staining was detectable in representative tissue blocks from both USPC. Strong cytoplasmic SAA positivity was evident in the positive control (i.e., liver).

To evaluate whether the high SAA expression detected by flow cytometry on primary USPC cell lines was compared with the expression of SAA of the USPC specimens from which the primary tumour cell lines were derived (i.e., USPC-ARK-1, USPC-ARK-2, and USPC-ARK-3) and/or whether *in vitro* expansion conditions may have modified protein expression, we evaluated SAA by immunohistochemical staining on formalin-fixed tumour tissue. As representatively shown in Figure 2 for USPC-ARK-1 and USPC-ARK-2, SAA was detected by IHC in all three USPC tissues. The intensity of staining for SAA was significantly higher among the tumour specimens compared with normal endometrial controls (*P*<0.001). Indeed, although all USPC tumours tested showed moderate (2+) or strong (3+) cytoplasmic positivity, normal control endometrial cells were found consistently negative for SAA expression whereas normal liver tissue (i.e., positive control) was found strongly positive for SAA expression (Figure 2).

### SAA secretion by primary USPC cell cultures

Primary short-term tumour cultures may provide an opportunity to study differential SAA secretion between highly enriched populations of tumour-derived epithelial cells. Cell-free supernatants from freshly isolated gynaecologic malignancies including three USPC and three squamous cervical carcinoma cell lines, as well as cultures of normal human fibroblasts and EBV cell lines, were collected and analysed for SAA expression levels by a sensitive bead-based immunoassay. Because prolonged passages *in vitro* are known to alter the physiology and phenotype of primary tumour cells, we performed all our experiments with highly purified tumour cells and normal cells (i.e., fibroblasts and EBV-transformed B cells) grown for less than 20 passages *in vitro*. Growth control medium was always analysed at the same time. In this regard, KSFM and RPMI-1640 media containing 10% fetal bovine serum had no detectable endogenous levels of SAA immunoreactivity (data not shown). All three primary USPC tumour cell lines tested secreted large amounts of SAA (mean=11.2 ng ml^−1^, range: 0.7–30.3 ng ml^−1^ per 10^5^ cells per 72 h). In contrast, undetectable to low secretion was identified in the supernatant of normal human fibroblasts (mean=0.12 ng ml^−1^), EBV-transformed B cells (mean=0.16 ng ml^−1^) or in those of three primary cervical carcinoma cell lines (i.e., not detectable) run in parallel (*P*<0.001).

### Serum SAA concentration in USPC and noncancer patients

**Figure 3:**
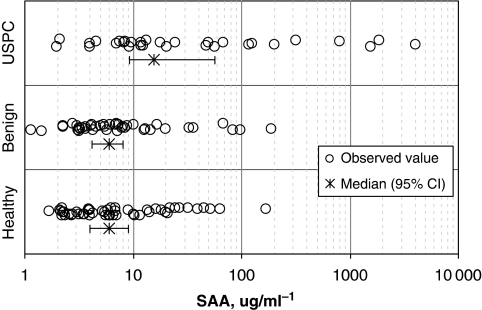
Distributions of serum SAA levels from 51 healthy subjects (bottom series), 42 benign-disease subjects (middle series), and 30 USPC patients (top series). Horizontal axis shows SAA in *μ*g ml^−1^. Circles show individual serum SAA levels, whereas ‘split-X’ symbols with error bars denote group medians with their rank-based 95% confidence intervals. The accompanying Table 4 contains the numerical values of medians, confidence intervals, and ranges, along with Mann–Whitney–Wilcoxon *P*-values and areas under ROC curves for the pairwise comparisons among the three groups.

**Figure 4:**
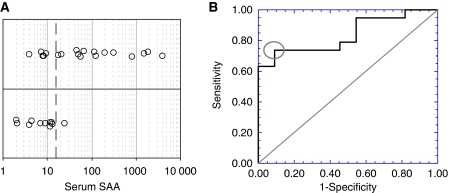
(**A**) Displays the distribution of serum SAA (*μ*g ml^−1^; horizontal axis) in high-stage (III-IV; top half) *vs* low-stage (I–II; bottom half) USPC patients. The heavy vertical dashed line denotes the median SAA (15.6 *μ*g ml^−1^) among all 30 patients. Fourteen of 19 high-stage patients had serum SAA above the median (74% sensitivity), whereas 10 of 11 low-stage patients had serum SAA below the median (91% specificity). (**B**) Displays the empirical Receiver Operating Characteristic (ROC) curve of the data in Panel **A**; the grey ellipse in Panel **B** circles the combination of sensitivity (74%) and specificity (91%) achieved using median SAA as a threshold to diagnose high-stage disease, and shows that this threshold maximises the sum of sensitivity and specificity in these patients. The area under the ROC curve was 0.837 (Mann–Whitney *P*=0.0024).

To investigate whether SAA is detectable in the serum of patients harbouring USPC, samples from 30 USPC patients, 51 healthy female controls and 42 women harbouring benign gynaecologic diseases were evaluated by a sensitive bead-based immunoassay (Millipore). SAA serum levels (*μ*g ml^−1^) from 51 healthy female controls had a median (95% CI) of 6.0 (4.0–8.9) and ranged from 1.6 to 169, whereas 42 patients with benign gynaecologic diseases had a median (95% CI) of 6.0 (4.2–8.1) and ranged from 1.1 to 189; their distributions were not statistically significantly different (area under ROC curve =0.506; *P*=0.92) (Table 2 and Figure 3). In contrast, serum SAA values from 30 USPC patients had a median (95% CI) of 15.6 (9.2–56.2) and ranged from 2.1 to 4000; these values were statistically significantly higher than those in the healthy group (area under ROC curve=0.732; *P*=0.0005) and benign group (area under ROC curve=0.739; *P*=0.0006). It is worth noting that in our series of USPC patients, all of whom were surgically staged, the majority were found to harbour advanced-stage disease (i.e., 63% stage III–IV). Thus, although most of our patients were considered to have clinical stage I disease before surgery, they were later up-staged at the time of comprehensive surgical staging laparotomy. In this regard, when preoperative SAA serum levels in USPC patients were analysed by stage, we found higher SAA serum levels in patients harbouring the most advanced USPC stages. Indeed, 14 (74%) of 19 USPC patients with Stage III or Stage IV disease had SAA levels above the 15.6-*μ*g ml^−1^ median, whereas 10 (91%) of 11 patients with Stage I or Stage II disease had SAA levels below the median (Figure 4A). These results correspond to 74% sensitivity and 91% specificity using the 15.6-*μ*g ml^−1^ median to distinguish advanced stage (III–IV) from early-stage (I–II) USPC. ROC analysis of serum SAA's staging potential (Figure 4B) showed that dichotomising SAA at or near 15.6 *μ*g ml^−1^ maximised the sum of sensitivity and specificity in this data set. The area under the ROC curve was 0.837 (*P*=0.0024). Importantly, high (>15.6 *μ*g ml^−1^) preoperative serum SAA values were predictive for advanced disease in 50% (4/8) of stage III patients and 91% (10/11) of stage IV patients with USPC.

## Discussion

USPC represents a variant of endometrial carcinomas characterised by a highly aggressive biological behaviour. The microscopic criteria for USPC diagnosis were first outlined by Hendrickson *et al* (1982). Classically, the neoplastic epithelium is characterised by serous differentiation with psammoma bodies present and with predominantly papillary architecture although solid areas can be focally detected (Hendrickson *et al*, 1982). Cytologically, pleomorphism, grade III nuclear atypia with prominent nucleoli and vesicular chromatin pattern, and a high mitotic activity are detected. Clinically, USPC has a propensity for early intraabdominal and lymphatic spread even at presentation (Hendrickson *et al*, 1982). Unlike the histologically indistinguishable serous ovarian carcinomas, USPC is a chemoresistant disease, because its onset with responses to combined cisplatinum-based chemotherapy is on the order of 20% and of short duration (Hendrickson *et al*, 1982; Goff *et al*, 1994; Carcangiu and Chambers, 1995; Chan *et al*, 2003). The survival rate is dismal, even when USPC is only a minor component of the histologically more common endometrioid adenocarcinoma, and widespread metastasis and death may occur even in those cases in which tumour is confined to the endometrium or to an endometrial polyp. The overall 5-year survival is about 30% for all stages, and the recurrence rate after surgery is extremely high (50–80%). The identification of biomarkers that can be used for early diagnosis, monitoring, and prediction of response to treatment in USPC might greatly contribute to the improvement of clinical management and outcomes of these patients. Unfortunately, no accepted and/or specific serum tumour markers have as yet been identified for this disease. In this regard, although it has been previously reported that the elevation of CA 125 level may be associated with an increase in the incidence of metastatic disease in endometrial tumours with endometrioid histology (Soper *et al*, 1990; Dotters, 2000), this marker appears to have limited utility in monitoring the effects of adjuvant therapy or in predicting tumour recurrence in USPC patients (Price *et al*, 1998). This report represents the first evaluation of SAA as a novel biomarker in USPC.

In this study, we have quantified SAA expression by RT–PCR in snap frozen USPC specimens. In addition, we have studied SAA protein expression and secretion in multiple primary gynaecologic malignancies including USPC and cervical cancer tumour cultures. We have confirmed the purity of the tumour cells in our short-term cultures by differential counts of Giemsa-stained cytospin slides as well as by cytokeratin expression using immunohistochemical techniques (data not shown). Our fresh tumour samples contained over 99% tumour cells. Finally, we have studied SAA levels in 123 serum samples derived from healthy donors, patients harbouring benign gynaecologic tumours and USPC patients.

We report for the first time a high level of expression of the SAA gene in USPC. Indeed, SAA gene expression was significantly higher in USPC when compared with NEC by RT–PCR. The average copy number of SAA-gene mRNA was 73 times higher in USPC compared with NEC cells. Consistent with these findings, highly purified primary USPC cultures were found positive for intracellular expression of SAA by flow cytometry as well as IHC and, importantly, were able to secrete high levels of SAA *in vitro* as detected by a sensitive bead-based immunoassay. In contrast, SAA was not detected in any of the three CVX cell lines tested and negligible levels were found in the culture supernatants of normal human fibroblasts cultures used as controls. Thus, taken together, our data highlighted for the first time a major tumour expression and secretion of SAA directly by USPC. More importantly, these results support our hypothesis that, in USPC patients, SAA is not only a liver-secreted protein but is also a USPC cell product. Of interest, Gutfeld *et al* (2006) have recently reported on SAA expression in normal, dysplastic, and neoplastic colonic mucosa. Using *in situ* hybridisation and IHC, they demonstrated the local and the differential expressions of SAA in human colon cancer tissues when compared with normal colonic mucosa. Furthermore they showed progressively higher SAA positivity through the different stages of dysplasia to overt carcinoma (Gutfeld *et al*, 2006). These findings in human colon carcinoma combined with our results in USPC seem to suggest a novel role for SAA autocrine production in colonic and endometrial tumorigenesis. In this regard, although the biological importance of SAA in USPC patients is not well understood, previous reports have suggested multiple important biologic functions of SAA. Indeed, SAA has been previously reported to be involved in cholesterol metabolism and transport, inhibition of lymphocyte, and endothelial cell proliferation, depression of the immune system, inhibition of platelet aggregation, and induction of adhesion, migration, and tissue infiltration of monocytes, neutrophils, lymphocytes, and mast cells (Malle *et al*, 2009).

Importantly, when SAA levels were quantified in the serum of USPC patients, we found elevated levels in USPC patients when compared with the levels found in healthy women. Furthermore, in the limited number of USPC patients where sequential serum samples were available (i.e., two patients), a decrease in SAA levels was observed post-operatively (data not shown). These *in vivo* data accord with our *in vitro* results from highly purified USPC primary cultures, and suggest that SAA is actively secreted by biologically aggressive USPC cells *in vitro*, and potentially *in vivo*. Importantly, we found no significant elevation of SAA in the serum of patients harbouring benign gynaecologic disease when compared with healthy female patients. We conclude that SAA may be a promising biomarker for early detection of recurrent USPC disease and for monitoring USPC response to adjuvant therapy.

It is worth noting that in our series of USPC patients, all of whom were surgically staged, the majority (63%) were found to harbour advanced disease. Thus, although most of our patients were considered to have clinical stage I disease before surgery, they were later up-staged at the time of comprehensive surgical staging laparotomy. Of interest, when preoperative SAA serum levels in our USPC patients were analysed by stage we found such levels to correlate with the stage of the disease (i.e., patients harbouring more surgically advanced stage disease that had significantly higher SAA levels when compared with patients harbouring stage I and stage II disease). High (>15.6 *μ*g ml^−1^) preoperative serum SAA values in patients otherwise clinically diagnosed with early-stage USPC were predictive for stage III and stage IV disease in 50% (four out of eight) and 91% (10 out of 11) of the USPC patients, respectively. Indeed, dichotomising serum SAA at or near 15.6 *μ*g ml ^−1^ achieved 74% sensitivity with 91% specificity for distinguishing advanced- (III–IV) from early-stage (I–II) disease. Thus, although larger studies that include more USPC patients harbouring surgically confirmed early- and late-stage disease will be necessary to confirm these findings, high SAA serum levels at the time of clinical diagnosis and before surgery may predict advanced-stage disease at the time of the staging laparotomy in the majority of patients. In this regard, it is important to point out that, because of the propensity of USPC to rapidly manifest extrauterine disease (i.e., positive lymph node metastases or spreading to the abdominal cavity), the USPC series reported here is likely most representative of the advanced stage disease commonly found in surgically staged USPC patients. In agreement with our data, Goff *et al* (1994) found 75% of patients with clinical stage I/II USPC to have extracorporeal disease when comprehensively staged. Similarly, Bristow *et al* demonstrated 74.4% of USPC patients to have advanced-stage disease following ovarian cancer-type surgical staging (Bristow *et al*, 2001). These findings were also corroborated in studies by O’Hanlan *et al* (1990) and Geisler *et al* (1999).

Several other potentially useful markers, including the human kallikrein enzymes hK6 and hK10 and cytokines such as interleukin-6, have recently been shown to be highly differentially expressed and secreted by USPC (Bellone *et al*, 2005; Santin *et al*, 2005a, 2006). It is thus possible that in analogy to what has recently been shown in ovarian cancer (Mor *et al*, 2005), the simultaneous evaluation of multiple markers such as SAA, hK6, hK10, and IL-6 by a multiplex, bead-based immunoassay system may ultimately allow the development of a test endowed with high specificity and sensitivity for the detection of USPC. This possibility is currently being investigated in our laboratory.

In conclusion, we report here the first evidence that SAA is highly expressed in USPC, it is actively secreted *in vitro*, and that high concentrations of SAA are present in the serum of USPC patients. Moreover, we have shown that high preoperative serum SAA levels in USPC patients clinically diagnosed with early-stage disease are predictive of more advanced stage disease at the time of comprehensive surgical staging. Our results strongly support the hypothesis that SAA may be used as a biomarker for this highly aggressive variant of endometrial cancer. Indeed, although SAA does not fulfil the criteria for an ‘ideal marker’, we believe that it has potential value in the initial assessment of patients harbouring USPC as well as in the potential monitoring of therapeutic results. The current availability of a highly sensitive and specific assay for measuring SAA protein concentration in serum, either alone or in combination with multiple additional biomarkers, will facilitate further studies to validate the clinical usefulness of the circulating levels of SAA for the management of patients with USPC.

## Figures and Tables

**Table 1:** Intracellular SAA expression in USPC and control cell lines

			**Binding of MAb**	
**Tumour line designation**	**Histology**	**Percentage of epithelial cells**	**Percentage of cells**	**MFI**
USPC-ARK1	Serous	>99	100	40
USPC-ARK2	Serous	>99	100	55
USPC-ARK3	Serous	>99	100	77
CVX-1	Squamous	>99	79	9
CVX-2	Squamous	>99	69	11
CVX-3	Squamous	>99	84	15
LCL-1	B cells	0	65	20
LCL-2	B cells	0	71	11

**Table 2:** Serum SAA in non-cancer (healthy), benign disease and USPC patients

**Subject group**	***N***	**Median (95% CI)^a^**	**Range^a^**
Healthy	51	6.0 (4.0–8.9)	1.6–169.2
Benign disease	42	6.0 (4.2–8.1)	1.1–189.9
USPC	30	15.6 (9.2–56.2)	2.1–4000
			
**Pairwise comparison**		**Area under ROC curve^b^**	***P*-value^c^**
Healthy *vs* benign		0.506	0.917
Healthy *vs* USPC		0.732	0.0005
Benign *vs* USPC		0.739	0.0006

a Medians, 95% confidence intervals, and ranges of serum SAA are in *μ*g ml^−1^.

b Values near 0.50 denote highly overlapping distributions; values near 1.00 denote highly separated distributions.

c *P*-values are from the two-sided Wilcoxon–Mann–Whitney test of the indicated comparison.
